# Analytical determination of theoretic quantities for multiple potential

**DOI:** 10.1038/s41598-020-73372-x

**Published:** 2020-10-16

**Authors:** C. A. Onate, M. C. Onyeaju, A. Abolarinwa, A. F. Lukman

**Affiliations:** 1 Department of Physical Sciences, Landmark University, Omu-Aran, Nigeria; 2 Theoretical Physics Group, Physics Department, University of Port Harcourt, Port Harcourt, Nigeria; 3 Department of Mathematics, University of Lagos, Akoka, Lagos State, Nigeria

**Keywords:** Information theory and computation, Quantum physics, Mathematics and computing, Physics

## Abstract

The approximate analytical solutions of the three-dimensional radial Schrödinger wave equation with a multiple potential function has been studied using a suitable approximation scheme to the centrifugal term in the framework of parametric Nikiforov–Uvarov method. The energy equation and the wave function were obtained. The calculated wave function was used to study Shannon entropy and variance via expectation values. The behaviour of Shannon entropy and variance respectively with the equilibrium bond length were examined in detail. A special case of the multiple potential (pseudoharmonic-like potential) was equally examined under Shannon entropy and variance. For further application of the study, some diatomic molecules were examined under variance and Shannon entropy. Finally, some variance inequalities were derived using Cramer-Rao uncertainty relation and these were justified by numerical results.

## Introduction

The essential reason for the probabilistic character of the quantum theory of physical system relies upon the uncertainty relation. This relation may be mathematically expressed by means of the Boltzmann-Shannon information entropy (entropic uncertainty relation) in a much more appropriate and accurate way than the standard deviation^1^. The information entropy is a superior measure of a spread and then of quantum uncertainty, a property of fundamental relevance for adequate characterization of position and momentum of single-particle densities^2^. These entropies have been used for various practical purposes such as the measurement of squeezing of quantum fluctuation^3^, reconstruction of charge and momentum densities of atomic and molecular systems^4^. In physical sciences, Shannon entropy measures the spread of the electron density. The concentration of the wave function of the state is higher when Shannon entropy is small^5^. Thus, Shannon entropy is used to determine the stability of a given system. A system is conceived to be more stable when Shannon entropy is small and becomes unstable when Shannon entropy is higher. Shannon entropy is related to fundamental and experimentally measurable quantities such as the kinetic energy and magnetic susceptibility which makes them useful in the study of the structure and dynamics of atomic and molecular systems^6^. It is noted that Shannon entropy has drawn many attentions due to its usefulness in different areas. Despite the various studies on Shannon entropy by different authors, Dehesa et al.^7^, stated that the analytical determination of the information entropies of physical systems is in its infancy. Recently, Yahya et al.^8^ and Onate et al.^9^ separately studied the Shannon entropy for some potential models using different traditional techniques. Motivated by this, we intend to investigate the analytical determination of Shannon entropy and Variance for a multiple potential which is proposed in the concept of the work. Another objective of this study is to derive some variance inequalities using the basic Cramer-Rao uncertainty relation of Fisher information.

**Figure 1:**
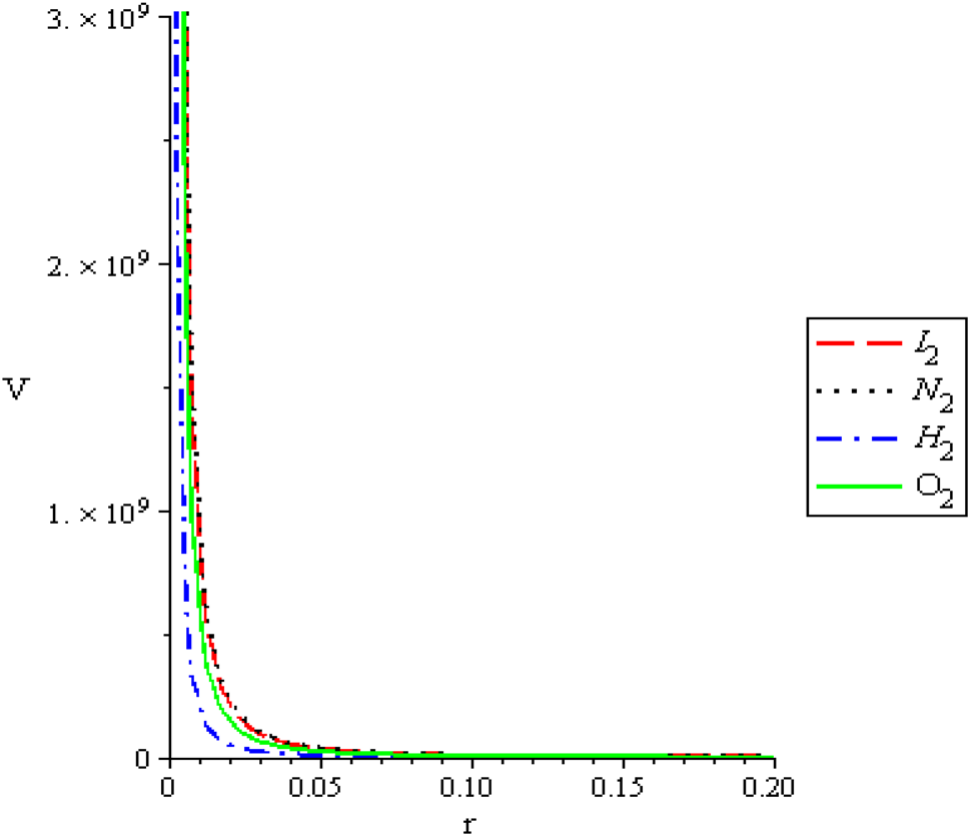
The behaviour of multiple potential with respect to $I_{2} ,N_{2} ,H_{2}$ and $O_{2}$ molecules.

The multiple potential is a combination of pseudoharmonic-like potential, double pure Coulomb potential and constant potential. The modification of the potential is to enable the potential fits in the study of some theoretic quantities with both spectroscopic and non-spectroscopic parameters. According to Sage and Goodisman^10^, the Pseudoharmonic potential is a useful potential. The authors pointed out that the harmonic oscillator potential (one of the most important potential model), is unrealistic in several aspect when compared to a real molecular vibrational potential, hence the suggestion for the Pseudoharmonic potential. According to them, the Pseudoharmonic potential maintains the availability of explicit solutions. This necessitate the inclusion of the Pseudoharmonic-like potential in this work. The multiple potential has a physical form

1 $$V(r) = \frac{{\left( {D_{e} r_{e}^{2} - C^{2} } \right)r_{e}^{2} + \left( {D_{e} + \lambda r^{2} r_{e}^{2} } \right)r^{2} - 2D_{e} r^{2} r_{e}^{2} }}{{r^{2} r_{e}^{2} }},$$

where $D_{e}$ is the dissociation energy, $r_{e}$ is the equilibrium bond length, $r$ is the internuclear separation, $C$ and $\lambda$ characterized the strengths of the potential. The change in their numerical values changes the shape of the potential. The multiple potential is a diatomic potential with the diatomic spectroscopic parameter given in Eq. (1). The Shape of the multiple potential and the Pseudoharmonic-like potential respectively, are shown in Fig. 1. The scheme of our presentation is as follows: “Method” section gives the solution of the radial equation with multiple potential. In “Some theoretic quantities and the multiple potential” section, we present the theoretic quantities. The discussion and conclusion are given in “Discussion of results” and “Conclusion” sections respectively.

## Method

### The radial Schrödinger equation and the multiple potential

In a spherical potential model $V(r),$ the time independent Schrödinger equation is given by^11–13^

2 $$\left( {\frac{{ - \hbar^{2} d^{2} }}{{2\mu dr^{2} }} + V(r) - \frac{{\ell (\ell + 1)\hbar^{2} }}{{2\mu r^{2} }}} \right)R_{n,\ell } (r) = E_{n,\ell } R_{n,\ell } (r),$$

where $\ell$ and $n$ are orbital angular momentum and radial quantum number respectively, $R_{n,\ell } (r)$ is the wave function, $\mu$ and $\hbar$ are reduced mass and Planck constant respectively, and $E_{n\ell }$ is the non-relativistic energy. Substituting Eq. (1) into Eq. (2), we have

3 $$\left[ {\frac{{d^{2} }}{{dr^{2} }} + \frac{2\mu }{{\hbar^{2} }}\left( {E_{n\ell } - \left[ {\frac{{\left( {D_{e} r_{e}^{2} - C^{2} } \right)r_{e}^{2} + \left( {D_{e} + \lambda r^{2} r_{e}^{2} } \right)r^{2} - 2D_{e} r^{2} r_{e}^{2} }}{{r^{2} r_{e}^{2} }}} \right] - \frac{{\hbar^{2} \ell (\ell + 1)}}{{2\mu r^{2} }}} \right)} \right]R_{n,\ell } (r) = 0.$$

Using the following transformation, $s = r^{2} ,$ Eq. (3) turns to

4 $$\left[ {\frac{{d^{2} }}{{ds^{2} }} + \frac{1}{2s}\frac{d}{ds} + \frac{{\frac{{ - \mu \left( {D_{e} + \lambda r_{e}^{2} } \right)s^{2} }}{{2\hbar^{2} r_{e}^{2} }} + \frac{{\mu \left( {E_{n,\ell } - D_{e} } \right)s}}{{2\hbar^{2} }} - \frac{\ell (\ell + 1)}{4} + \frac{{\mu \left( {D_{e} r_{e}^{2} - C^{2} } \right)}}{{2\hbar^{2} }}}}{{S^{2} }}} \right]R_{n,\ell } (s) = 0.$$

Comparing Eq. (4) with equation (A1), have the values for the parametric constants in equation (A4) as follows

5 $$\left. \begin{aligned} \alpha_{1} & = \frac{1}{2},\alpha_{2} = \alpha_{3} = 0,\alpha_{4} = \frac{1}{4},\alpha_{5} = 0,\alpha_{6} = \frac{{\mu \left( {D_{e} + \lambda r_{e}^{2} } \right)}}{{2\hbar^{2} r_{e}^{2} }},\alpha_{7} = - \frac{{\mu \left( {E_{n,\ell } - D_{e} } \right)}}{{2\hbar^{2} }}, \\ \alpha_{8} & = \frac{1}{16} + \frac{\ell (\ell + 1)}{4} + \frac{{\mu \left( {D_{e} r_{e}^{2} - C^{2} } \right)}}{{2\hbar^{2} }},\alpha_{9} = \frac{{\mu \left( {D_{e} + \lambda r_{e}^{2} } \right)}}{{2\hbar^{2} r_{e}^{2} }},\alpha_{10} = 1 + \sqrt {\left( {1 + 2\ell } \right)^{2} + \frac{{8\mu \left( {D_{e} r_{e}^{2} - C^{2} } \right)}}{{\hbar^{2} }}} , \\ \alpha_{11} & = \sqrt {\frac{{2\mu \left( {D_{e} + \lambda r_{e}^{2} } \right)}}{{\hbar^{2} r_{e}^{2} }}} ,\alpha_{12} = \frac{1}{4} + \frac{1}{4}\sqrt {\left( {1 + 2\ell } \right)^{2} + \frac{{8\mu \left( {D_{e} r_{e}^{2} - C^{2} } \right)}}{{\hbar^{2} }}} ,\alpha_{13} = - \sqrt {\frac{{2\mu \left( {D_{e} + \lambda r_{e}^{2} } \right)}}{{\hbar^{2} r_{e}^{2} }}} , \\ \end{aligned} \right\}.$$

Substituting Eq. (5) into equation (A2) and equation (A3) respectively, we have the energy equation and the corresponding wave function as

6 $$E_{n,\ell } = 2D_{e} + \frac{\hbar }{{\mu r_{e} }}\sqrt {\frac{{\mu \left( {D_{e} + \lambda r_{e}^{2} } \right)}}{2}} \left( {4n + \frac{3}{2} + \sqrt {\left( {1 + 2\ell } \right)^{2} + \frac{{8\mu \left( {D_{e} r_{e}^{2} - C^{2} } \right)}}{{\hbar^{2} }}} } \right),$$

7 $$R_{n,\ell } (s) = N_{n,\ell } s^{{\frac{1}{4} + \frac{1}{4}\sqrt {\left( {1 + 2\ell } \right)^{2} + \frac{{8\mu \left( {D_{e} r_{e}^{2} - C^{2} } \right)}}{{\hbar^{2} }}} }} e^{{ - \sqrt {\frac{{\mu \left( {D_{e} + \lambda r_{e}^{2} } \right)}}{{2\hbar^{2} r_{e}^{2} }}} s}} L_{n}^{{\frac{1}{2}\sqrt {\left( {1 + 2\ell } \right)^{2} + \frac{{8\mu \left( {D_{e} r_{e}^{2} - C^{2} } \right)}}{{\hbar^{2} }}} }} \left( {2\sqrt {\frac{{\mu \left( {D_{e} + \lambda r_{e}^{2} } \right)}}{{2\hbar^{2} r_{e}^{2} }}} s} \right),$$

where $N_{n,\ell }$ is a normalization constant which can be determine using normalization condition below

8 $$\int\limits_{0}^{\infty } {\left| {R_{n,\ell } (r)} \right|^{2} } dr = 1.$$

The normalization constant is thus obtain as

9 $$N_{n,\ell } = \left( {\frac{{n!2\left( {2\sqrt {\frac{{\mu \left( {D_{e} + \lambda r_{e}^{2} } \right)}}{{2\hbar^{2} r_{e}^{2} }}}^{2} } \right)^{{\frac{1}{4}\left( {2\sqrt {\left( {1 + 2\ell } \right)^{2} + \frac{{8\mu \left( {D_{e} r_{e}^{2} - C^{2} } \right)}}{{\hbar^{2} }}} + 3} \right)}} }}{{\Gamma \left( {n + \sqrt {\left( {1 + 2\ell } \right)^{2} + \frac{{8\mu \left( {D_{e} r_{e}^{2} - C^{2} } \right)}}{{\hbar^{2} }}} + \frac{3}{2}} \right)}}} \right)^{\frac{1}{2}} .$$

### Some theoretic quantities and the multiple potential

In this section, we calculate some theoretic quantities such as Shannon entropy and Variance. The quantities are calculated using the probability density function which is the square of the wave function.

#### Expectation values

In this section, we calculate the expectation values of the multiple potential. To obtain the radial expectation values, we use the Hellmann Feynman theory^14–18^. If the Hamiltonian for a particular quantum system is a function of the parameter $V$, then, taking the eigenvalues as $E_{n,\ell } (V)$ and eigenfunction as $R_{n,\ell } (V)$ of the Hamiltonian, we can write from Hellmann Feynman Theory that

10 $$\frac{{\partial E_{n,\ell } (V)}}{\partial V} = \left\langle {R_{n,\ell } (V)\left| {\frac{\partial H(V)}{{\partial V}}} \right|R_{n,\ell } (V)} \right\rangle ,$$

provided that $R_{n,\ell }$ is continuous with respect to $V.$ In terms of our potential, the effective Hamiltonian is given as

11 $$H = - \frac{{\hbar^{2} }}{2\mu }\frac{{d^{2} }}{{dr^{2} }} + \frac{{\ell (\ell + 1)\hbar^{2} }}{{2\mu r^{2} }} + \frac{{\left( {D_{e} + \lambda r^{2} r_{e}^{2} } \right)r^{2} }}{{r^{2} r_{e}^{2} }} + \frac{{D_{e} r_{e}^{2} - C^{2} - 2D_{e} r^{2} }}{{r^{2} }}.$$

When $V = D_{e}$ and $V = \mu ,$ then the expectation values of $r^{2}$ and $p^{2}$ for multiple potential are obtain as follows

12 $$\left\langle {r^{2} } \right\rangle = \frac{1}{2}\sqrt {\frac{{\hbar^{2} r_{e}^{2} }}{{2\mu \left( {D_{e} + \lambda r_{e}^{2} } \right)}}} \left( {2 + 4n + \sqrt {\left( {1 + 2\ell } \right)^{2} + \frac{{8\mu \left( {D_{e} r_{e}^{2} - C^{2} } \right)}}{{\hbar^{2} }}} } \right),$$

13 $$\left\langle {p^{2} } \right\rangle = \sqrt {\frac{{\mu \hbar^{2} D_{e}^{2} }}{{2\left( {D_{e} r_{e}^{2} + \lambda } \right)}}} \left( {2 + 4n + \sqrt {\left( {1 + 2\ell } \right)^{2} + \frac{{8\mu \left( {D_{e} r_{e}^{2} - C^{2} } \right)}}{{\hbar^{2} }}} } \right) - \frac{{16D_{e} \mu^{3} \sqrt {\frac{{D_{e} r_{e}^{2} + \lambda }}{{\hbar^{2} }}} }}{{\sqrt {\left( {1 + 2\ell } \right)^{2} + \frac{{8\mu \left( {D_{e} r_{e}^{2} - C^{2} } \right)}}{{\hbar^{2} }}} }}.$$

#### Shannon entropy

Shannon entropy measures the spread of electron density and as such, it is used to determine the system’s stability. As pointed out earlier, the smaller the Shannon entropy, the more concentration of the wave function of the state. In this work, Shannon entropy will be considered in both position space and momentum space. In the position space, Shannon entropy is given as ^9,19–22^

14 $$S(\rho ) = - 4\pi \int\limits_{0}^{\infty } {r^{2} \rho (r)In\rho (r)dr} ,$$

where $\rho (r)$ is the probability density function that can be obtained from the radial wave function. For the position space, the radial wave function given in Eq. (6) can be written as

15 $$R_{n,\ell } (r) = N_{n,\ell } r^{\eta } e^{{ - \eta r^{2} }} L_{n}^{\eta - 1} \left( {2\eta r^{2} } \right),$$

where

16 $$\eta = \sqrt {\left( {1 + 2\ell } \right)^{2} + \frac{{8\mu \left( {D_{e} r_{e}^{2} - C^{2} } \right)}}{{\hbar^{2} }}} .$$

The probability density function is the squared of the radial wave function. Thus, the probability density is given

17 $$\rho (r) = N_{n,\ell }^{2} r^{2\eta } e^{{ - 2\eta r^{2} }} \left[ {L_{n}^{\eta - 1} \left( {2\eta r^{2} } \right)} \right]^{2} .$$

Thus, with Eq. (17), Eq. (14) becomes

18 $$S(\rho ) = \frac{3}{2}\left[ {In\left( {\frac{n}{2\chi }} \right) - \eta R} \right] + \eta R\left[ {\frac{1}{R}In(n) - n - \eta } \right] - \Phi + In(2\pi ) + o(1),$$

where

19 $$\Phi = In\left( {\frac{2n!}{{\Gamma \left( {n + \eta + \frac{3}{2}} \right)}}} \right),$$

20 $$\chi = \sqrt {\frac{{\mu \left( {D_{e} + \lambda r_{e}^{2} } \right)}}{{2\hbar^{2} r_{e}^{2} }}} .$$

In the momentum space, Shannon entropy is given as

21 $$S(\gamma ) = - 4\pi \int\limits_{0}^{\infty } {p^{2} \gamma (p)In\gamma (p)dp} ,$$

where $\gamma (p)$ is the probability density function for the momentum space, the radial wave function given in Eq. (7) can be written as

22 $$R_{n,\ell } (p) = N_{n,\ell } p^{\eta } e^{{ - \frac{{p^{2} }}{\chi }}} L_{n}^{\eta - 1} \left( {\frac{{p^{2} }}{2\chi }} \right).$$

The probability density then becomes

23 $$\gamma (p) = N_{n,\ell }^{2} p^{2\eta } e^{{ - \frac{{2p^{2} }}{\chi }}} \left[ {L_{n}^{\eta - 1} \left( {\frac{{p^{2} }}{2\chi }} \right)} \right]^{2} .$$

With the probability density in Eq. (23), the Shannon entropy for momentum space in Eq. (21) becomes

24 $$S(\gamma ) = \frac{3}{2}\left[ {In(n \times 2\chi ) + \eta R} \right] + \eta R\left[ {\frac{1}{R}In(n) + n + \eta } \right] - \Phi + In(2\pi ) + o(1),$$

From the work of Dehesa et al.^22^, it can be shown for an accurate result that at the ground state,

25 $$S(\rho ) + S(\gamma ) \ge (1 + log\pi ).$$

This will be verified later in the numerical results.

#### Variance

In this section, we calculate the variance of a multiple potential. Given a normalized-to-unity (probability) density $\rho (r)$, there are several functional quantities for measuring the uncertainty or information content associated with the density. Among the three popular ones is the variance which is given as^23,24^

26 $$V(\rho ) = \int {\left( {r - \left\langle r \right\rangle_{\rho } } \right)^{2} = \left\langle {r^{2} } \right\rangle_{\rho } - } \left\langle r \right\rangle_{\rho }^{2} .$$

Equation (26) above can be solved using expectation values. However, Dehesa et al.^19^, showed that a relationship exist between Fisher Information and Variance. These authors pointed out that the Fisher Information in momentum space is equal to four times the variance in position space. Thus,

27 $$I(\gamma ) = 4V(\rho ).$$

These authors also showed the relationship between the expectation values and Fisher Information as

28 $$I(\gamma ) = 4\left\langle {p^{2} } \right\rangle .$$

Comparing Eqs. (27) and (28), we can write the variance for position space as

29 $$V(\rho ) = \left\langle {p^{2} } \right\rangle .$$

Following the work of Dehesa et al.^19^, the variance for momentum space can be written as

30 $$V(\gamma ) = \left\langle {r^{2} } \right\rangle .$$

At this juncture, we want to derive some uncertainty inequalities for variance based on Eqs. (27), (28), (29) and (30). To begin the derivation, we first considered the upper bound. For any $n$ and $\ell ,$ the upper bound for the position and momentum spaces of the variance is given as

31 $$V(\rho )V(\gamma ) \ge \left\langle {p^{2} } \right\rangle \left\langle {r^{2} } \right\rangle .$$

From Cramer-Rao inequalities,

32 $$I(\rho ) \ge \frac{9}{{\left\langle {r^{2} } \right\rangle }},$$

33 $$I(\gamma ) \ge \frac{9}{{\left\langle {p^{2} } \right\rangle }}.$$

From Eqs. (32) and (33), we can now establish the inequality

34 $$16V(\gamma )V(\rho ) \ge \frac{81}{{\left\langle {p^{2} } \right\rangle \left\langle {r^{2} } \right\rangle }}.$$

Combining Eqs. (31) and (34) leads to another inequality of the form

35 $$V(\gamma )V(\rho ) \ge \frac{9}{4}.$$

The authenticity of Eqs. (34) and (35) will be verified in the numerical results as will be seen in subsequent tables.

## Discussion of results

Table 1 showed the spectroscopic parameters for some diatomic molecules studied in this work. In Table 2, we presented the numerical values of the explicit bound state energies. The numerical results of our calculation showed that the results obtained with $- C = \lambda ,$ $C = 0$ and $\lambda = 0,$ are equivalent. The results of the analytical determination of information entropies calculated for the ground state in both position space and momentum space are shown in various Tables. In Table 3, we numerically verify the uncertainty relation in Eq. (25). The Shannon entropy in the position space is bounded while in the momentum space the Shannon entropy is unbounded for the various values of the dissociation energy of multiple potential. The physical meaning of the inequality is that a decrease in Shannon entropy for position space corresponds to an increase in Shannon entropy for momentum space. This indicates that a diffused density distribution $\gamma (p)$ in momentum space is associated with a localized density distribution $\rho (r)$ in the position space or configuration space. It can be seen that the lower bound for the sum of the Shannon entropy is 13.18210163 which is greater than $(1 + log\pi )$ that is equal to 1.497206180. However, the position space Shannon entropy exhibit squeezing effect in Table 3. However, the numerical results in this Table satisfied Bialynick-Birula, Mycielski inequality.

**Table 1:** Model parameters used for diatomic molecules^25^.

Molecules	$\mu \left( {{\text{amu}}} \right)$	$r_{e}$ (Å)	$D_{e} \left( {{\text{cm}}^{ - 1} } \right)$
${\text{O}}_{2} \left( {X^{3} \sum_{g}^{ + } } \right)$	7.997457504	1.207	42,041
${\text{I}}_{2} \left( {XO_{g}^{ + } } \right)$	63.45223502	2.666	12,547
${\text{H}}_{2} \left( {X^{1} \sum_{g}^{ + } } \right)$	0.50407	0.741	38,318
${\text{N}}_{2} \left( {X^{1} \sum_{g}^{ + } } \right)$	7.00335	1.097	79,885

**Table 2:** Bound state energy eigenvalues for different quantum number with $\mu =\lambda =C=\mathcal{l}=\mathcal{\hslash }={r}_{e}=1.$

*n*	*D*_*e*_ = 3, $\lambda = 0.1$	*D*_*e*_ = 3, $\lambda = 0.1$	*D*_*e*_ = 3.1, $C = - 0.1$
	$- C = \lambda$	$C = 0$	$\lambda = 0$
0	3.010733500	3.019407758	2.905492953
1	7.990693340	7.999367597	7.885452792
2	12.97065318	12.97932744	12.86541263
3	17.95061302	17.95928728	17.84537247
4	22.93057286	22.93924211	22.82533231
5	27.91053270	27.91920695	27.80529215

**Table 3:** Numerical results for the uncertainty relation $S(\rho ) + S(\gamma ) \ge (1 + log\pi )$ at the ground state.

$D_{e}$	$S(\rho )$	$S(\gamma )$	$S(\rho ) + S(\gamma )$
1	− 5.948747623	19.13084786	13.18210163
2	− 12.44873487	27.63076352	15.18202865
3	− 20.94874562	38.13077459	17.18202897
4	− 32.48872864	51.67054871	19.18182007
5	− 44.98872356	66.17048667	21.18176311

In Table 4, we presented numerical values for variance in position space and momentum space for ground state and the first excited state. The position and momentum spaces for the variance have an inverse relationship with each other. A strongly localized distribution in the momentum space corresponds to widely delocalized distribution in the position space. The results obtained in each case, followed the same trend and obeyed Heisenberg Uncertainty Principle. To test the variance-inequalities presented in Eqs. (34) and (35) respectively, we generated numerical results in Table 5. Following Eq. (34), it can be seen that sixteen multiplied by the product of variance cannot go lower than eight one divided by the product of the expectation values. The lower bound for $16V(\rho )V(\gamma )$ is 36.66666667 while the upper bound for $81/\left\langle {p^{2} } \right\rangle \left\langle {r^{2} } \right\rangle$ is 35.34545454. Hence, Eq. (34) is justified. Similarly, Eq. (35) shows that the product of variance cannot go lower than nine divided by four. The lower bound for the product of variance is 2.291666667 while nine divided by four is 2.250000000. This also justified the variance inequality in Eq. (35).

**Table 4:** Variance in position space and momentum space for the ground state and the first excited state with $\mu = \lambda = C = \ell = \hbar = r_{e} = 1$ for five different values of *D*_*e*_*.*

*D*_*e*_	*n* = 0		*n* = 1	
	$$V\left( \rho \right)$$	$$V\left( \gamma \right)$$	$$\mathrm{V}(\uprho )$$$$V\left( \rho \right)$$	$$\mathrm{V}(\upgamma )$$$$V\left( \gamma \right)$$
1	1.833333333	1.250000000	3.833333333	2.250000000
2	4.385922817	1.249873702	7.651909141	2.066370283
3	6.887964888	1.237436867	11.13060557	1.944543648
4	9.318517831	1.224522872	14.37816209	1.856974040
5	11.69571702	1.212886510	17.46921971	1.790236779

**Table 5:** Numerical results for variance uncertainty relations $16V(\rho )V(\gamma ) \ge 81/\left\langle {p^{2} } \right\rangle \left\langle {r^{2} } \right\rangle$ and $V(\rho )V(\gamma ) \ge 9/4$ at the ground state.

$D_{e}$	$V(\rho )$	$V(\gamma )$	$V(\rho )V(\gamma )$	$16V(\rho )V(\gamma )$	$81/\left\langle {p^{2} } \right\rangle \left\langle {r^{2} } \right\rangle$
1	1.833333333	1.250000000	2.291666667	36.66666667	35.34545454
2	4.385922817	1.249873702	5.481849588	87.70959341	14.77603475
3	6.887964888	1.237436867	8.523421691	136.3747471	9.503225692
4	9.318517831	1.224522872	11.41073822	182.5718115	7.098576660
5	11.69571702	1.212886510	14.18557740	226.9692384	5.710024888

Finally, we presented numerical values of variance both in momentum space and position space in Tables 6 and 7 respectively for some selected diatomic molecules using the spectroscopic parameters in Table 1. These Tables showed that the increase in both the quantum number and angular momentum quantum number for the multiple potential do not satisfied uncertainty principle in terms of variance. In Tables 8 and 9, we numerically presented the Shannon entropy for position space and momentum space respectively of four molecules with various $\lambda .$ The position space and momentum space information entropies have an inverse relationship with each other. A strongly localized distribution in the momentum space corresponds to widely delocalized distribution in the position space. However, an entropy squeezing is noted for the position space. In the two Tables, there is a change in position space and momentum space Shannon entropies as the parameter $\lambda ,$ goes up but the sum is bounded above the inequality for BBM. In Table 10, we numerically studied Shannon entropy in position space and momentum space for different values of $C$ for $\ell = 0$ and $\ell = 1.$ For the two values of $\ell ,$ the Shannon entropy in the position space is bounded while in the momentum space the Shannon entropy is unbounded for multiple potential. It can be seen that the lower bound for the sum of the Shannon entropy with $\ell = 0$ is 4.916111226 while the lower bound for $\ell = 1$ is 11.49671887. In each case, the lower bound for the sum of the entropies is greater than $(1 + log\pi )$ that is equal to 1.497206180. Thus, a general formulation of information theoretic uncertainty relations for the different conjugate pair of observables is described for some parameters and all dimensions for multiple potential as the inequality satisfied Bialynick-Birula, Mycielski inequality. It is observed that the Shannon entropy in position space exhibit entropy squeezing which is contrary to the behaviour of Shannon entropy in momentum space. In Table 11, we examined the variation of variance with various values of $\lambda$ and $C$ respectively. It is seen that the variance in momentum space for both cases exhibit squeezing effect. However, the position space and momentum space variance obeyed uncertainty relation.

**Table 6:** Variance in momentum space of some diatomic molecules with $\lambda = C = 1$ for various *n* and $\ell$.

*n*	$\ell$	O_2_	N_2_	I_2_	H_2_
0	0	1.713683194	1.396006143	5.982996805	1.387897058
1	0	2.285682117	1.802891599	6.650268750	2.867617323
	1	2.341869074	1.837106595	6.669938147	3.450327279
2	0	2.857681039	2.209776975	7.317540695	4.347337587
	1	2.913867996	2.243992012	7.337210092	4.930047543
	2	3.020397590	2.309707703	7.376345357	5.623959059
3	0	3.429679961	2.616662392	7.984812641	5.827057851
	1	3.485866918	2.650877428	8.004482037	6.409767808
	2	3.592296512	2.716593119	8.043617502	7.103679323
	3	3.739761789	2.809335511	8.101822709	7.822629830

**Table 7:** Variance in position space of some diatomic molecules with $\lambda = C = 1$ for various *n* and $\ell$.

*n*	$\ell$	O_2_	N_2_	I_2_ ${\mathrm{I}}_{2}$	H_2_
0	0	2245.249263	3433.300487	13,276.17490	61.35717001
1	0	2998.59`309	4436.789214	14,758.22574	134.0880400
	1	3073.035594	4521.441144	14,801.95574	165.9774063
2	0	3751.933354	5440.277941	16,240.27659	206.8189099
	1	3826.377640	5524.929871	16,284.00658	238.7082763
	2	3967.304321	5687.478173	16,371.01363	274.1176200
3	0	4505.275400	6443.766668	17,722.32743	279.5497798
	1	4579.719686	6528.418598	17,766.05743	311.4391462
	2	4720.646367	6690.966900	17,853.06448	346.8484900
	3	4915.756753	6920.284223	17,982.46578	382.8143814

**Table 8:** Shannon entropy in position space of some diatomic molecules with $n = \ell = \lambda = 1.$

$\lambda$	O_2_	N_2_	I_2_	H_2_
1	− 9.366561662	− 10.11718503	− 9.858501175	− 8.129260503
2	− 9.797355994	− 10.47016837	− 10.37182813	− 8.476578674
3	− 10.06924975	− 10.70922703	− 10.67373644	− 8.713050458
4	− 10.26837488	− 10.89022332	− 10.88840033	− 8.892565198
5	− 10.42557063	− 11.03592104	− 11.05509862	− 9.037302968
6	− 10.55545988	− 11.15786673	− 11.19139989	− 9.158576082
7	− 10.66614005	− 11.26272994	− 11.30669852	− 9.262941848
8	− 10.76256756	− 11.35471379	− 11.40661120	− 9.354542870
9	− 10.84799788	− 11.43663873	− 11.49476497	− 9.436164065

**Table 9:** Shannon entropy in momentum space of some diatomic molecules with $n = \ell = \lambda = 1.$

$\lambda$	O_2_	N_2_	I_2_	H_2_
1	20.86636363	21.95086858	20.90105286	19.94808018
2	21.29715796	22.30385191	21.41437981	20.29539835
3	21.56905172	22.54291057	21.71628812	20.53187013
4	21.76817685	22.72390686	21.93095201	20.71138487
5	21.92537260	22.86960458	22.09765030	20.85612264
6	22.05526185	22.99155027	22.23395157	20.97739576
7	22.16594202	23.09641348	22.34925021	21.08176152
8	22.26236953	23.18839734	22.44916288	21.17336254
9	22.26236953	23.27032227	22.53731666	21.25498374

**Table 10:** Shannon entropy in both position space and momentum space with $$n = 0,C = D_{e} = 1,$$$$r_{e} = 0.2$$ with two values of the angular momentum quantum number.

$C$	$S(\rho )$	$S(\gamma )$	$S(\rho ) + S(\gamma )$
$$\ell = 0$$			
0.05	− 4.640958227	10.58925839	5.948300163
0.10	− 4.544309000	10.41809255	5.873783547
0.15	− 4.380101535	10.12571650	5.745614967
0.20	− 4.142456423	9.698844067	5.556387645
0.25	− 3.819485125	9.110802974	5.291317849
0.30	− 3.383145160	8.299256386	4.916111226
$$\ell = 1$$			
0.05	− 14.61649333	26.51582516	11.89933183
0.10	− 14.54857673	26.41382482	11.86524809
0.15	− 14.43528304	26.24355239	11.80826935
0.20	− 14.27645948	26.00459066	11.72813118
0.25	− 14.07188417	25.69633589	11.62445173
0.30	− 13.82125748	25.31797636	11.49671887

**Table 11:** Variance in both position space and momentum space for various $$\lambda$$ and $$C$$ with $$n = \ell = 1,$$$$D_{e} = 10$$ and $$r_{e} = 1.2$$.

$C$	$V(\gamma )$	$V(\rho )$	$\lambda$	$V(\gamma )$	$V(\rho )$
0.5	2.139242226	− 26.06961441	0.5	2.174306340	− 26.55625199
1.0	2.104772267	− 28.01273932	1.0	2.104772267	− 28.01273932
1.5	2.045281346	− 31.54798261	1.5	2.041509182	− 29.42994916
2.0	1.957067601	− 37.27753145	2.0	1.983627833	− 30.81043801
2.5	1.833118858	− 46.55925627	2.5	1.930406053	− 32.15651621
3.0	1.658889662	− 63.07534762	3.0	1.881250446	− 33.47027854

**Figure 2:**
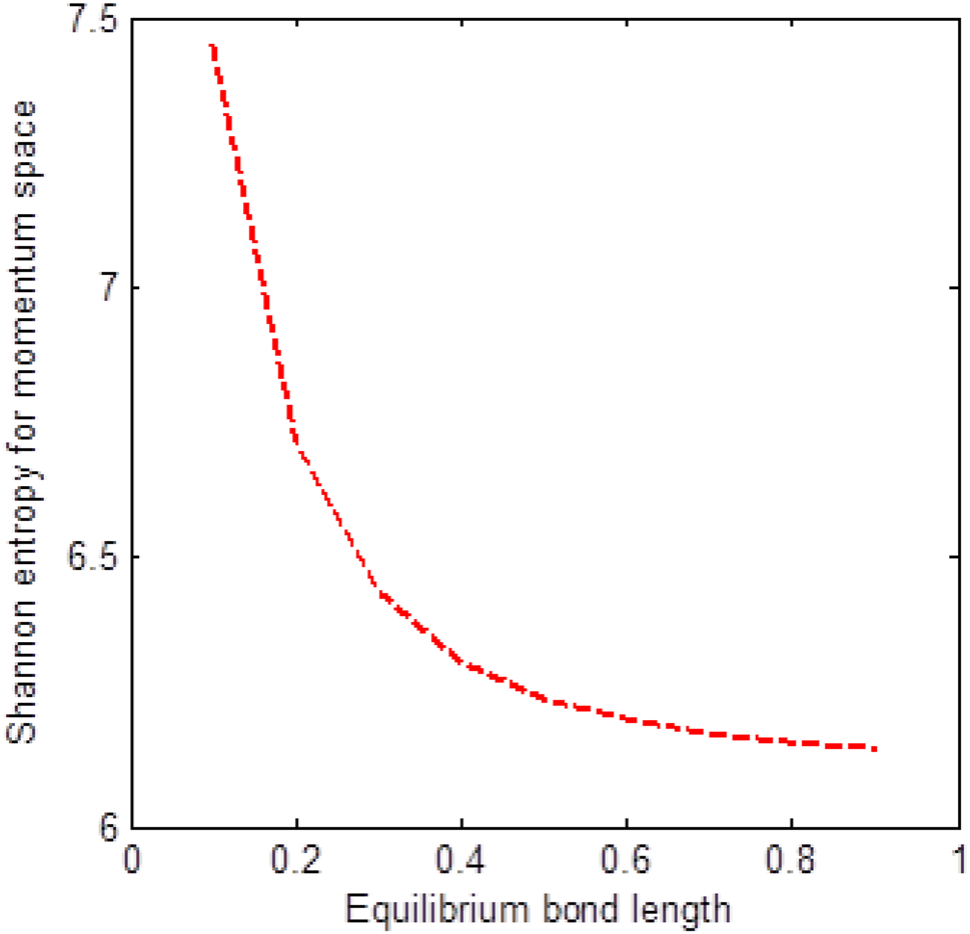
Shannon entropy for momentum space against the equilibrium bond length with $\mu = \ell = \hbar = 1,$ $n = D_{e} = C = 1,$ and $\lambda = 2C$ of the multiple potential functions.

**Figure 3:**
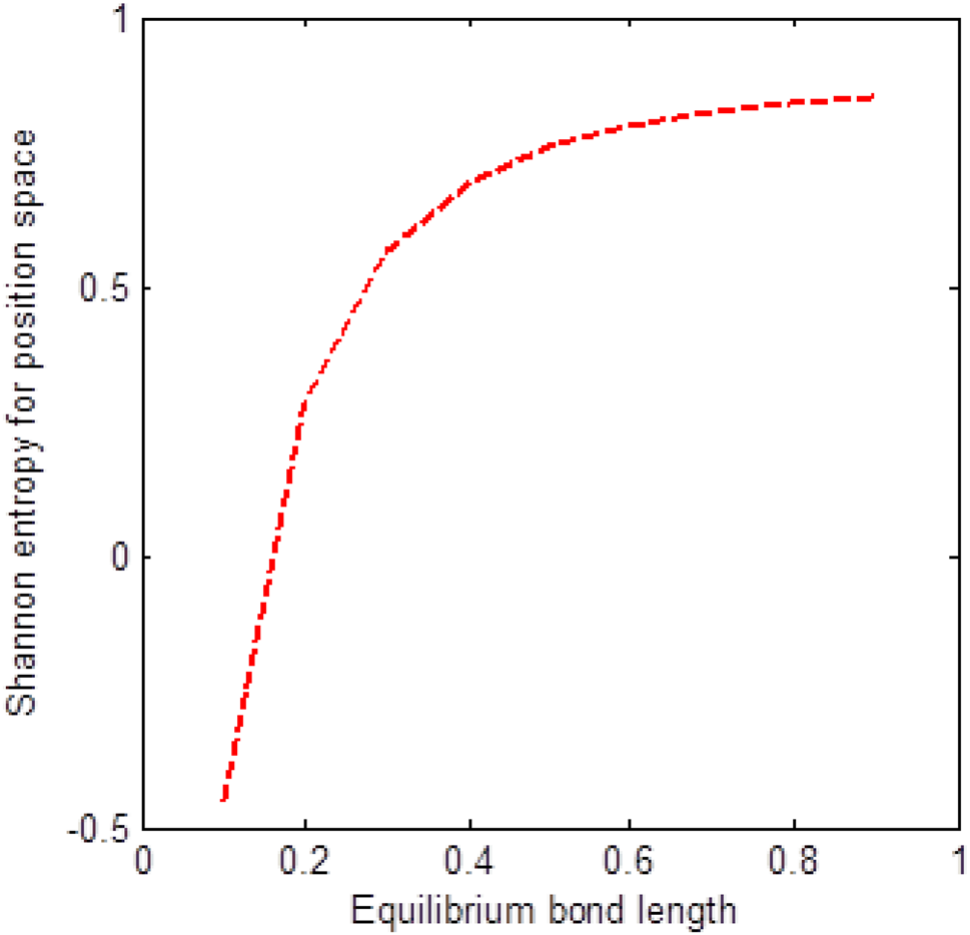
Shannon entropy for position space against the equilibrium bond length with $\mu = \ell = \hbar = 1,$ $n = D_{e} = \lambda = 1,$ and $C = \lambda$ of the multiple potential functions.

**Figure 4:**
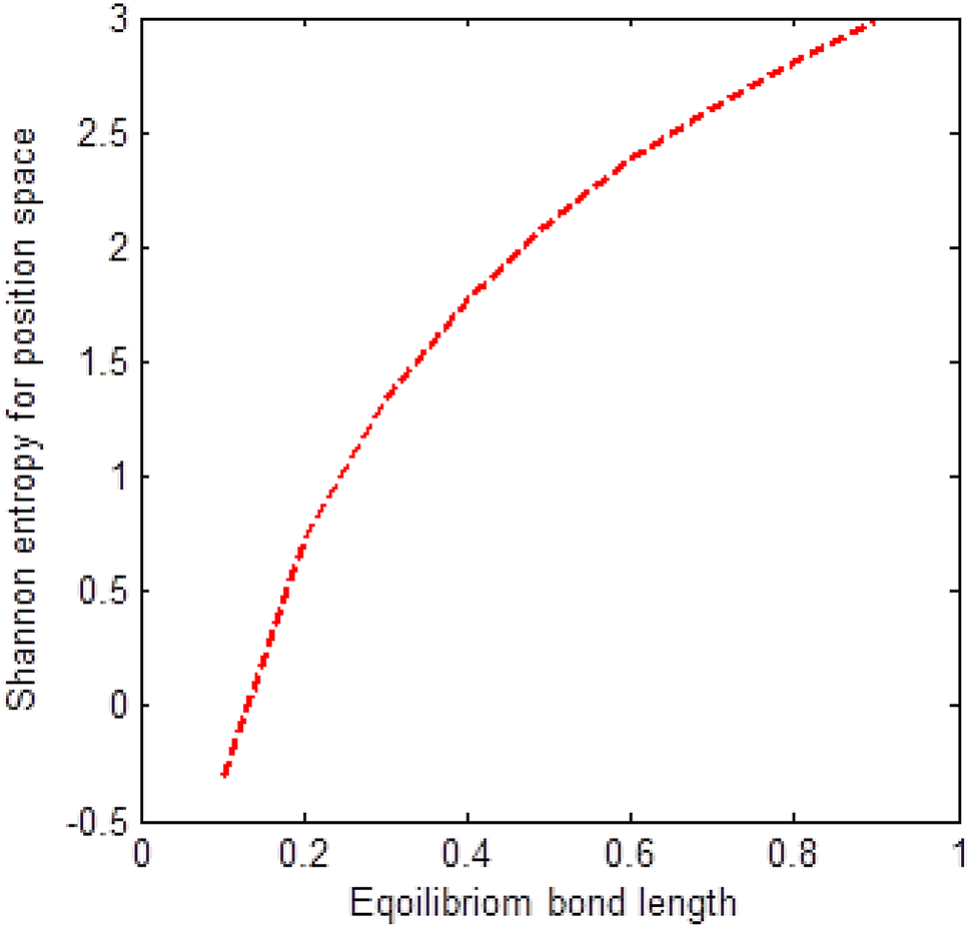
Shannon entropy for position space against the equilibrium bond length with $\mu = n = \ell = \hbar = D_{e} = \lambda = 1,$ and $C = 0$ of the pseudoharmonic-like potential functions.

**Figure 5:**
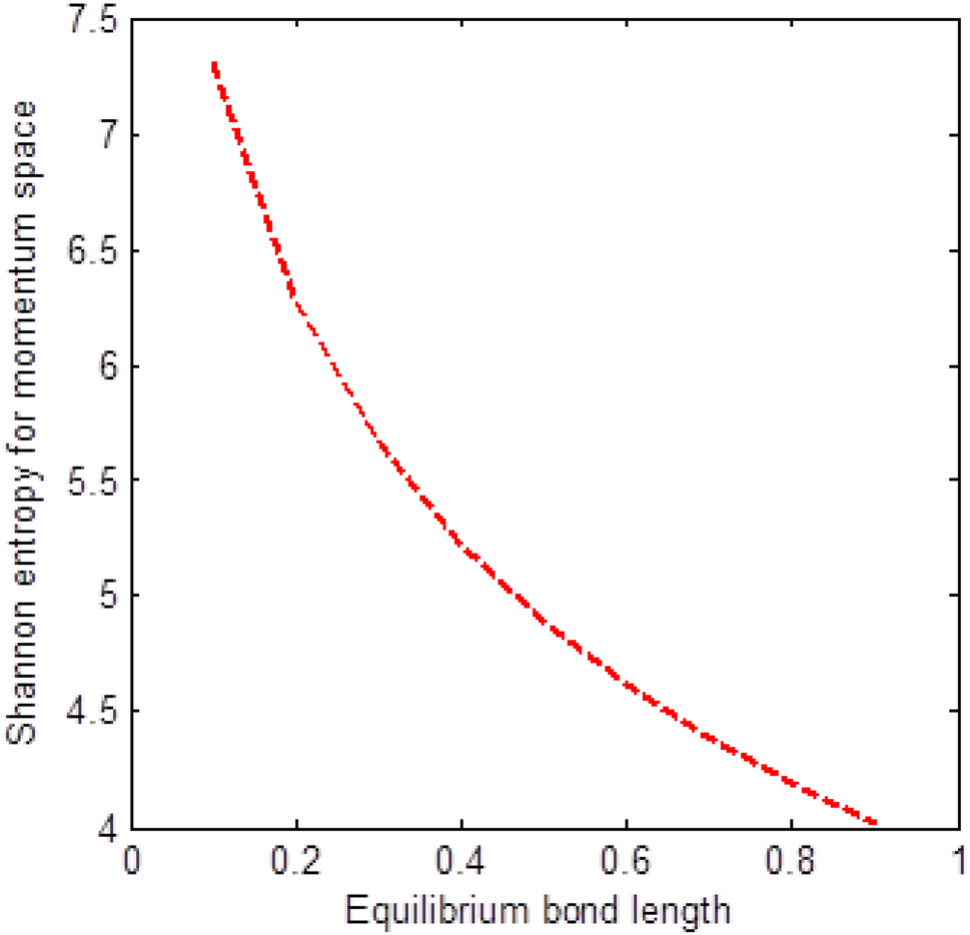
Shannon entropy for momentum space against the equilibrium bond length with $\mu = n = \ell = \hbar = D_{e} = \lambda = 1,$ and $C = 0$ of the pseudoharmonic-like potential functions.

**Figure 6:**
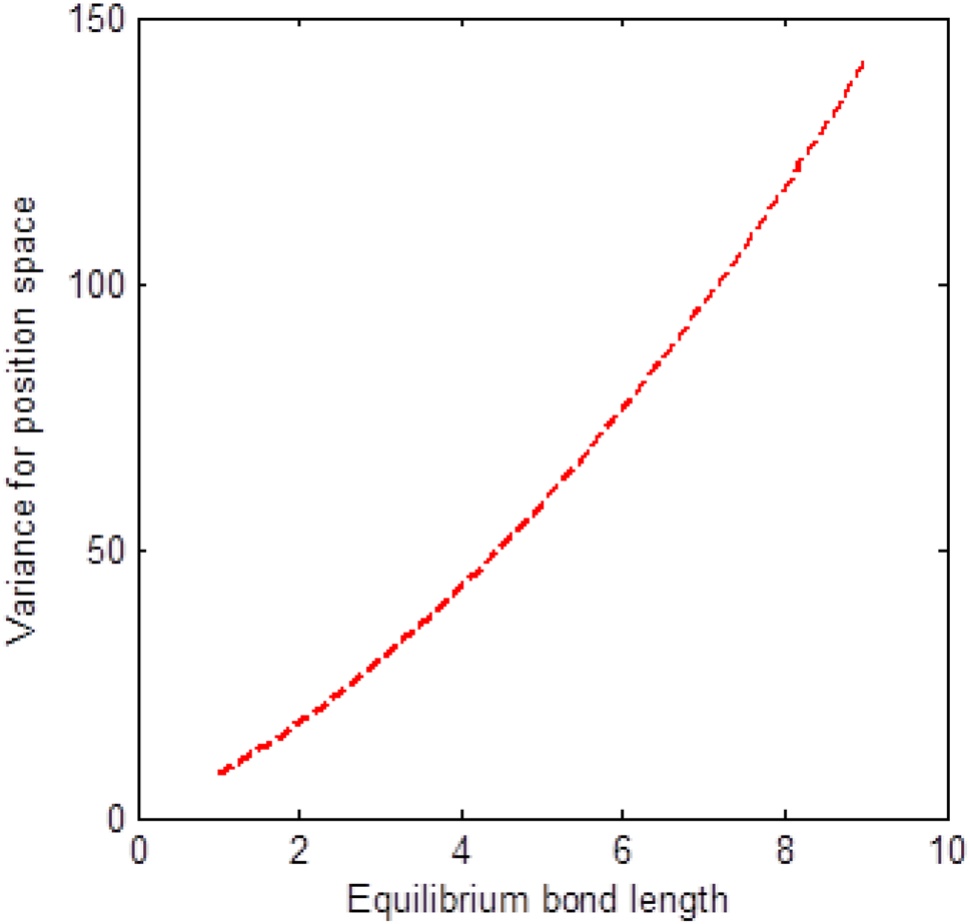
Variance for position space against the equilibrium bond length with $\mu = n = \ell = \hbar = D_{e} = C = 1,$ and $\lambda = 2C$ of the multiple potential functions.

**Figure 7:**
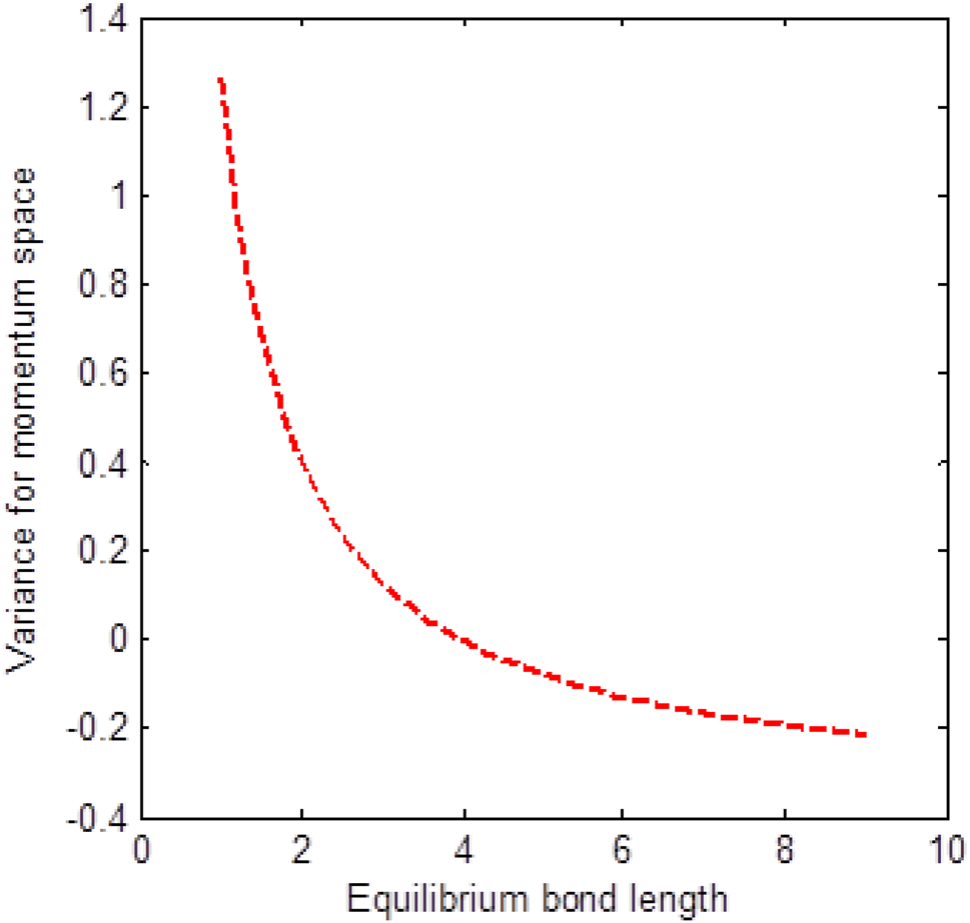
Variance for momentum space against the equilibrium bond length with $\mu = n = \ell = \hbar = D_{e} = C = 1,$ and $\lambda = 2C$ of the multiple potential functions.

**Figure 8:**
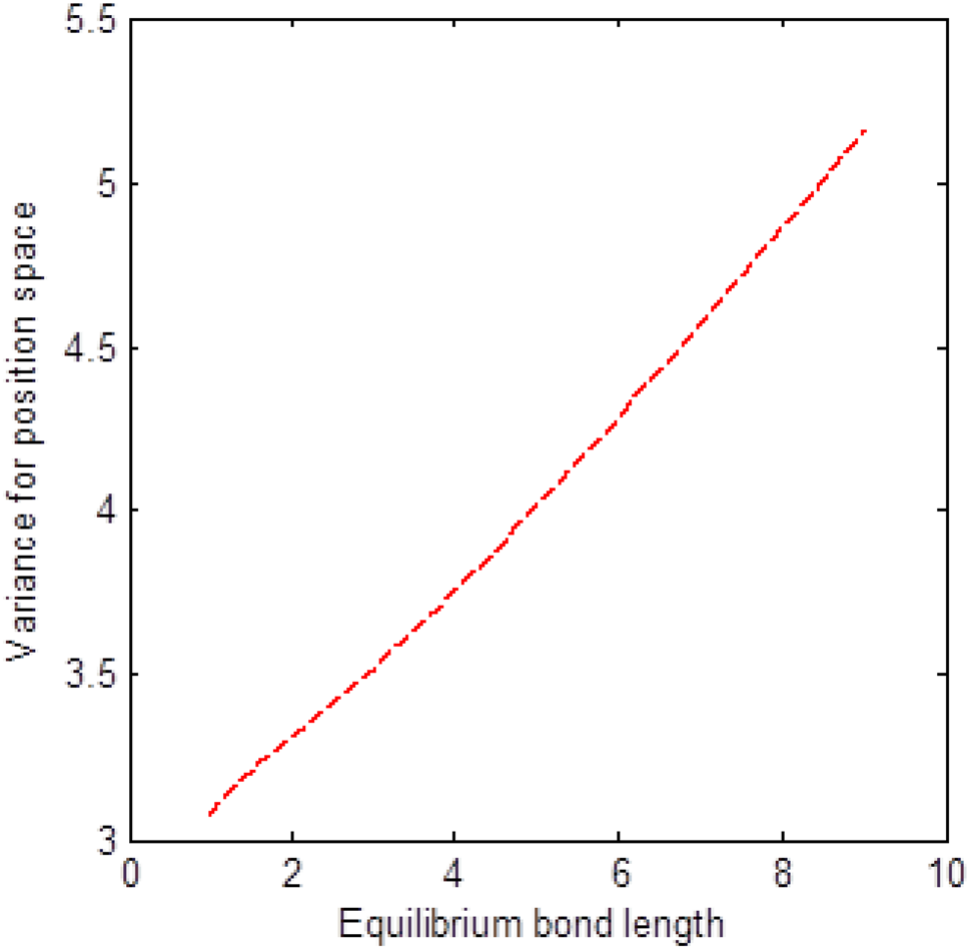
Variance for position space against the equilibrium bond length with $\mu = n = \ell = \hbar = D_{e} = \lambda = 1,$ and $C = 0$ of the pseudoharmonic-like potential function.

**Figure 9:**
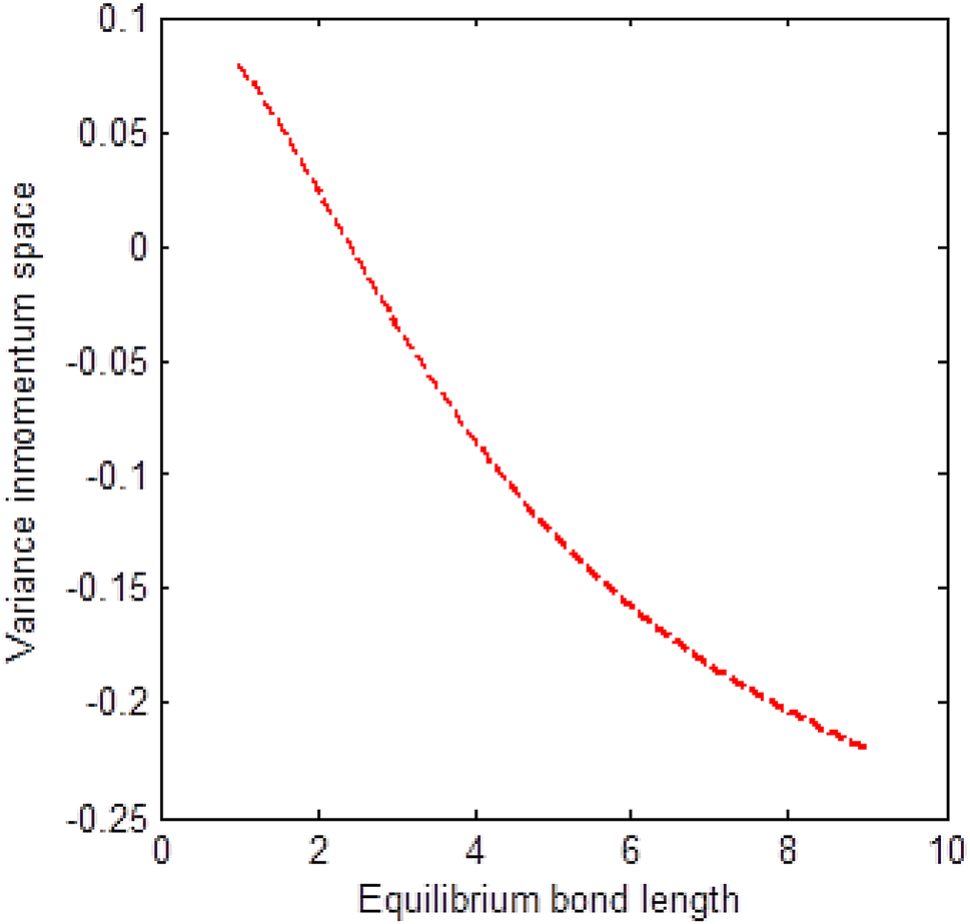
Variance for momentum space against the equilibrium bond length with $\mu = n = \ell = \hbar = D_{e} = \lambda = 1,$ and $C = 0$ of the pseudoharmonic-like potential function.

**Figure 10:**
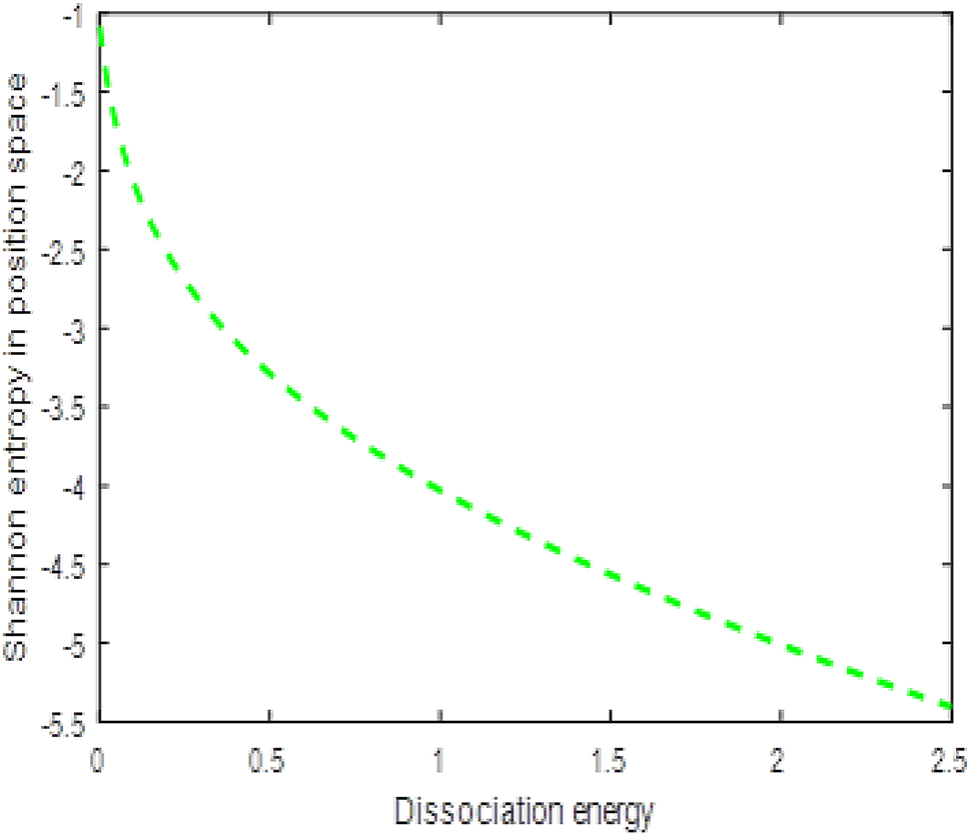
Shannon entropy for position space against the dissociation energy with $\mu = n = \ell = \hbar = C = \lambda = 1$ and $r_{e} = 0.2$ of the multiple potential function.

**Figure 11:**
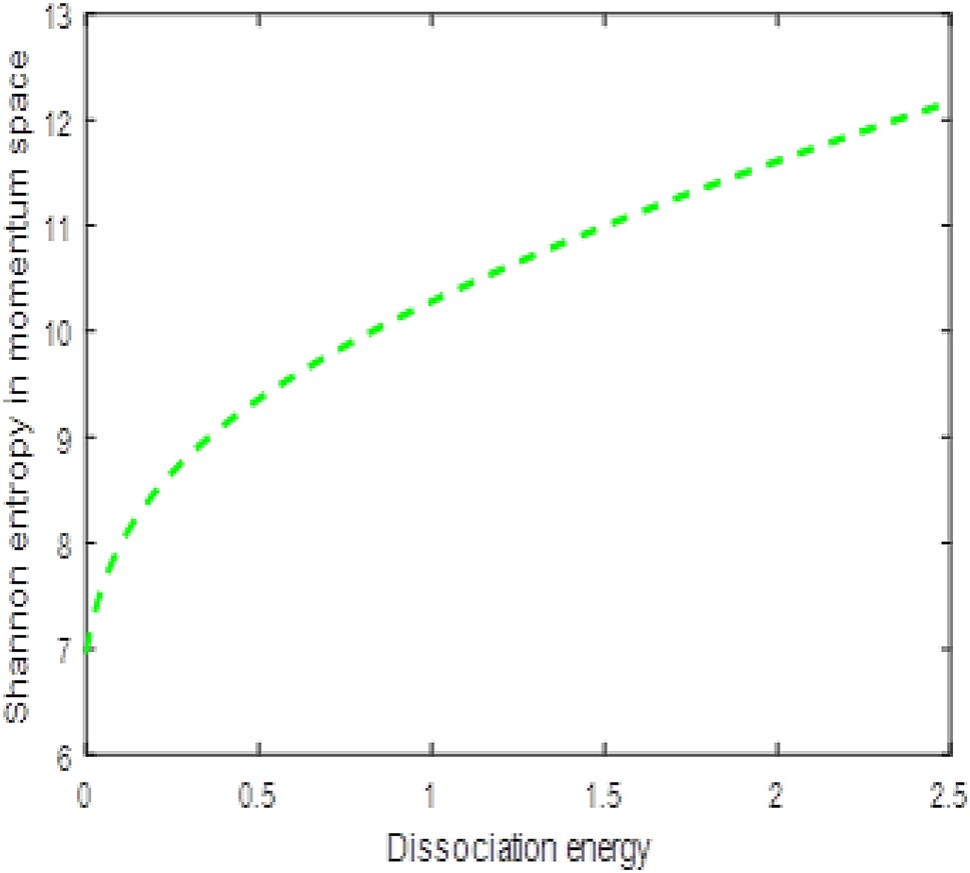
Shannon entropy for momentum space against the dissociation energy with $\mu = n = \ell = \hbar = C = \lambda = 1$ and $r_{e} = 0.2$ of the multiple potential function.

The shape of multiple potential and pseudoharmonic-like potential respectively are shown in Fig. 1. In Figs. 2 and 3, we plotted Shannon entropy for momentum space and position space respectively against the equilibrium bond length for the multiple potential functions. In the momentum space, as the equilibrium bond length increases linearly, there is a sharp monotonic decrease in the Shannon entropy while in position space, there is a sharp increase in the Shannon entropy as the equilibrium bond length increases. However, Shannon entropy experience more squeezing in Fig. 3. As the value of the equilibrium bond length increases, the Shannon entropies tends towards a constant value. In Figs. 4 and 5, we plotted Shannon entropy for position space and momentum space respectively against the equilibrium bond length for a pseudoharmonic-like potential. The results obtained is equivalent to the results of the multiple potential. Figures 6 and 7 showed the variation of variance for both position space and momentum space respectively against the equilibrium bond length for multiple potential. In the position space, variance increases as the equilibrium bond length increases while in the momentum space, an increase in the equilibrium bond length results to a monotonic decrease in variance. There is more squeezing effect in the momentum space as the equilibrium bond length increases. The same results were observed in Figs. 8 and 9 for pseudoharmonic-like potential. In Fig. 9, the variance for momentum space becomes squeezing. The Shannon entropy for position space and momentum space respectively, are plotted against the dissociation energy in Figs. 10 and 11. In Fig. 10, as the dissociation energy increases from zero, there is more concentration of the wave function of the state which results to more stability of the system. In this Fig, the Shannon entropy for position space becomes squeezing as the dissociation energy increases linearly. In Fig. 11 however, there is less concentration of the wave function of the state as the dissociation energy goes up. This brings about less stability of the system.

## Conclusion

We have used the parametric Nikiforv-Uvarov method to obtain the analytic solutions of the the radial Schrödinger equation for multiple potential. Our results showed that the numerical values of the multiple potential are equivalent to the numerical values of the two subset potentials studied. Some expectation values were calculated using Hellmann–Feynman theorem. A new variance uncertainty relation established has been justified by numerical results. Finally, a Shannon entropy uncertainty and inequality relation was also confirmed by generating numerical values using MATLAB 9.2.0.538062. Our results revealed that the Shannon entropy for various $\lambda$ with $\ell = 0$ do not satisfied Bialynick-Birula, Mycielski inequality while with $\ell = 1$ satisfied it.

## Supplementary information

Supplementary information.
