# Supplementary material for: Analytical determination of theoretic quantities for multiple potential
Source: Sci Rep. 2020 Oct 16;10:17542. doi: 10.1038/s41598-020-73372-x (PMC7568580; doi:10.1038/s41598-020-73372-x)
Supplement: Supplementary file 1 — Supplementary information. [file 41598_2020_73372_MOESM1_ESM.docx]

**APPENDIX**

**Parametric Nikiforov-Uvarov Method.**

The parametric Nikiforov-Uvarov method is one of the shortest and accurate traditional techniques to solve bound state problems. This method was derived from the conventional Nikiforov-Uvarov method by Tezcan and Sever [27]. According to Tezcan and Sever, the reference equation or standard equation for the parametric Nikiforov-Uvarov is

$\left(\frac{d^{2}}{dr^{2}}+\frac{\alpha_{1}-\alpha_{2}}{\mathrm{s}(1-\alpha_{3}s)}\frac{d}{ds}+\frac{-\xi_{1}s^{2}+\xi_{2}s-\xi_{3}}{s^{2}(1-\alpha_{3}s)^{2}}\right)\psi(s)=0.$ (A1)

Following the work of Tezcan and Sever, the condition for eigenvalues and eigenfunction are respectively given by [27, 28, 29, 30, 31]

$n\alpha_{2}-\left(2n+1\right)\alpha_{5}+\alpha_{7}+2\alpha_{3}\alpha_{8}+n\left(n-1\right)\alpha_{3}+\left(2n+1\right)\sqrt{\alpha_{9}}+\left(2\sqrt{\alpha_{9}}+\alpha_{3}\left(2n+1\right)\right)\sqrt{\alpha_{8}}=0,$ (A2)

 (A3)

The parametric constants in equations (3) and (4) are deduced as follows

$\left.\begin{matrix} \alpha_{4}=\frac{1-\alpha_{1}}{2},\alpha_{5}=\frac{\alpha_{2}-2\alpha_{3}}{2},\alpha_{6}=\alpha_{5}^{2}+\xi_{1},\alpha_{7}=2\alpha_{4}\alpha_{5}-\xi_{2},\alpha_{8}=\alpha_{4}^{2}+\xi_{3}, \\ \alpha_{9}=\alpha_{3}\left(\alpha_{7}+\alpha_{3}\alpha_{8}\right)+\alpha_{6},\alpha_{10}=\alpha_{1}+2\alpha_{4}+2\sqrt{\alpha_{8}},\alpha_{11}=\alpha_{2}-2\alpha_{5}+2\left(\sqrt{\alpha_{9}}+\alpha_{3}\sqrt{\alpha_{8}}\right), \\ \alpha_{12}=\alpha_{4}+\sqrt{\alpha_{8}},\alpha_{13}=\alpha_{5}-\left(\sqrt{\alpha_{9}}+\alpha_{3}\sqrt{\alpha_{8}}\right) \end{matrix}\right\}.$ (A4)

Reference

[27] Tezcan, C. & Sever, R. A General Approach for the Exact Solution of the Schrödinger Equation. *Int. J. Theor. Phys*. **48,** 337-350 (2009).

[28] Onate, C.A. Approximate Solutions of the Non-Relativistic Schrӧdinger Equation with

Pӧschl-Teller Potential. *Chin. J. Phys*. **53,** 060002 (2015).

[29] Hamzavi, M., Thylwe, K.E. & Rajabi, A.A. Approximate Bound States Solution of the Hellmann Potential*. Commun. Theor. Phys*. **60,** 1-8 (2013).

[30] Setare, M.R. & Haidari, S. Spin symmetry of the Dirac equation with the Yukawa potential. *Phys. Scr*. **81**, 065201 (2010).

[31] Zarrinkar, S., Rajabi, A.A., Hassanabadi, H. & Rahimov, H. [Analytical treatment of the two-body spinless Salpeter equation with the Hulthen potential](javascript:void(0)). *Phys. Scr.* **84,** 0065008 (2011).
